# Group Membership and Social and Personal Identities as Psychosocial Coping Resources to Psychological Consequences of the COVID-19 Confinement

**DOI:** 10.3390/ijerph17207413

**Published:** 2020-10-12

**Authors:** Carlos-María Alcover, Fernando Rodríguez, Yolanda Pastor, Helena Thomas, Mayelin Rey, José Luis del Barrio

**Affiliations:** 1 Department of Psychology, Universidad Rey Juan Carlos, 28922 Alcorcón, Madrid, Spain; fernando.rodriguez@urjc.es (F.R.); yolanda.pastor@urjc.es (Y.P.); helena.thomas@urjc.es (H.T.); 2 Centro de Salud Mental de Ciudad Lineal, Hospital Universitario Ramón y Cajal, 28017 Madrid, Spain; mayelin.rey@salud.madrid.org; 3 Department of Medical Specialties and Public Health, Universidad Rey Juan Carlos, 28922 Alcorcón, Madrid, Spain; jose.delbarrio@urjc.es

**Keywords:** group membership, social identity, social support, mental health and well-being, COVID-19 pandemic

## Abstract

The confinement imposed by measures to deal with the COVID-19 pandemic may in the short and medium term have psychological and psychosocial consequences affecting the well-being and mental health of individuals. This study aims to explore the role played by group membership and social and personal identities as coping resources to face the experience of the COVID-19 confinement and radical disruption of social, work, family and personal life in a sample of 421 people who have experienced a month of strict confinement in the Region of Madrid. Our results show that identity-resources (membership continuity/new group memberships, and personal identity strength) are positively related to process-resources (social support and perceived personal control), and that both are related to better perceived mental health, lower levels of anxiety and depression, and higher well-being (life satisfaction and resilience) during confinement. These results, in addition to providing relevant information about the psychological consequences of this experience, constitute a solid basis for the design of psychosocial interventions based on group memberships and social identity as coping resources.

## 1. Introduction

The accelerated expansion of COVID-19 in the early months of 2020 forced governments to enact extraordinary and emergency measures of social distancing to deal with the pandemic. As a consequence, millions of people were suddenly isolated and confined to their households. In March 2020, in just one week large cities such as Milan, Madrid or New York underwent rigid limitations on mobility and going out on the street, attending non-essential work and normal activities of daily life were forbidden. In the following days, the number of people infected and dead from COVID-19 increased in fairly fast progression. Overflowing hospitals, intensive care units at risk of collapse, overwhelmed health authorities and shortages of health resources to protect health professionals and citizens, generated a state of alarm unknown to current generations in developed countries. Investigating and understanding how people dealt with a situation without referents is essential to identify resources that can be useful in an emergent and global crisis.

There are antecedents of pandemic situations related to contagious diseases (i.e., the epidemics of SARS 2002–2003, the pandemic 2009 H1N1, or the Ebola virus disease epidemic 2013–2016) [1], natural disasters and humanitarian crises caused by war conflicts. However, the scopes, large-scale and sudden nature of the COVID-19 pandemic in a globalized world, make it an unprecedented experience for most of humanity. Consequently, we need data that sheds light on the mechanisms and processes that people mobilize and develop to coping experiences that, directly and vicariously, dramatically affect their lives and, eventually, their physical and mental health.

Disaster research pointed out the importance of going beyond psychological coping approaches. Thus, a perspective that considers coping as a social process includes a collective dimension, an experience-based dimension, and a local dimension, highlighting resources such as social capital and community resilience [2]. Social Identity Theory (SIT) [3] argues that in any social context people define themselves from their group membership, which means define their self in terms of social identity. If the multiple groups that define the self “provide a person with stability, meaning, purpose, and direction, then this will typically have positive implications for that individual’s mental health” ([4], p. 5). Extensive research from the Social Cure approach [5] has provided robust evidence on the importance of social factors, specifically of the curative role played by social integration and social support, for mental and physical health [5].

Based on these approaches, this study aims first, to explore the possible existence of significant differences in relation to several socio-demographic and occupational variables regarding group memberships, social and personal identities, social support and health and well-being factors during the COVID-19 pandemic. And second, to explore the role played by group membership, social and personal identities and social support as coping resources to face the experience of the COVID-19 confinement and radical disruption of social, work, family and personal life. To do this, it uses a sample of 421 people who have experienced a month of strict confinement in the Region of Madrid. In mid-July, Spain had the third most deaths proportionally to their COVID-19 cases for population in the world (60.80 deaths for 100,000 population, behind Belgium, 85.69, and United Kingdom, 67.76) [6]; and Madrid was one of the European cities with the highest incidence of contagion (1071 per 100,000 population) and deaths (126 per 100,000 population; 8418 people have died (29.7% of deaths in Spain), suffered between March and June 2020.

### Theoretical Background and Research Questions

The rapid global expansion since the end of 2019 in Wuhan, in the Chinese province of Huabei, of the Coronavirus named COVID-19, led to the majority of the European governments decreeing confinement, quarantine and isolation measures in their own homes or in public dependencies (hospitals, hotels, ocean liners, etc.). These measures affected millions of people on a scale never previously known. Confinement is considered a public health preventive measure to reduce the risk of disease transmission, and affects not only sick people, but also healthy or asymptomatic individuals. Quarantine involves separating and restricting the movement of people who have potentially been exposed to the contagious disease to determine if they are ill or have symptoms; their goal is to reduce the risk that they could infect other people in their close or public social context. And isolation refers to the social separation of people who have already been diagnosed with the contagious disease, so that they are not in contact with non-sick people [7,8]. Despite these differences between public health measures, the terms are often used interchangeably, and in practice people subjected to them by the authorities experience diverse thoughts and emotions due to the limitation or loss of freedom of movement, doubts or fear of being infected (themselves or their loved ones and acquaintances), the loss or reduction of interpersonal and social contact, the disruption of daily habits both at work and leisure, boredom, stigma, and uncertainty about the future [7,9,10].

There is scientific evidence of the psychological and psychosocial consequences of the confinement, quarantine and isolation measures studied in recent infectious diseases, such as SARS, N1H1, or Ebola [7,8,11,12,13,14], and the current COVID-19 in China [15]. These consequences are usually related to problems of anxiety, negative affectivity, irritability, fear, frustration, panic, depression and other negative emotional reactions, leading to infected people experiencing stigma and suicidal ideation or intentions. In turn, these consequences can lead to other health problems, both physical and mental, and a reduction in the quantity and quality of sleep. Overall, literature review suggests [7,10] that the psychological impact of quarantine and confinement is wide-ranging, substantial, detrimental, and can be long lasting, depending on how long it lasts; evidence shows that a longer quarantine is associated with poorer psychological outcomes. However, this evidence has been obtained basically from an individual perspective, or a clinical approach, and as far as we are aware, the role played by collective and social factors in the psychological consequences of confinement and quarantine has not been sufficiently investigated.

The social world is the canvas where human lives are painted. Given the social nature of human beings, group membership and high-quality social relationships are vital for health and well-being. Social contact and belonging to many groups are fundamental and pervasive human motivations [16] and shape the roots of personal and social identity.

For decades, research has accumulated data and solid arguments that show an association between social relationships and health [17]. Social factors play a central role in shaping health outcomes, and its effects are similar and often larger than that of poor health habits [5]. Less socially isolated or more socially included people had better psychological and physical health and less likely to die, at least prematurely [17]. Large meta-analytic data across 308,849 individuals, followed for an average of 7.5 years, was conclusive [18]. Individuals with adequate levels in two social factors associated with interpersonal relationships (social inclusion and social support) have a 50% greater likelihood of survival compared to those with poor or sparse social relationships. In an additional meta-analysis across 70 independent studies in which several possible confounders were statistically controlled for, the weighted average effect sizes corresponded to an average of increased likelihood of mortality by 29% for social isolation, 26% for loneliness, and 32% for living alone [19].

Social inclusion occurs primarily through group membership. Being a member of multiple groups satisfies the need to belong and, especially, provides identities [16]. SIT theory has demonstrated the central role of groups in building the social self. Furthermore, individual self-concept includes both personal identity and group memberships, since people need others to validate their attitudes and behaviors [20]. As Tajfel [21] has pointed out, social identity is the part of self-concept that derives from their awareness of belonging to social groups, as well as from the emotional and evaluative meaning associated with these multiple memberships. In sum, SIT [5,22] has stressed that social behavior depends largely on the degree to which people see others sharing their social identities with them.

Internalized group memberships provide shared social identities. In turn, important psychological resources with implications for health are derived from shared social identities [5]. These psychological resources primarily refer to perceptions of social connection and positive orientation to other people; a sense of meaning, purpose, self-worth through social connectedness and group life; social support exchange with people who define themselves in terms of shared social identity; and the development of a sense of control, efficacy and power [5,23]. Extensive research has shown that one of the key resources that flow from shared social identities and group memberships are social support exchange opportunities, that is, interaction contexts for receiving, giving, and benefit from both social actions [5,24,25]. Empirical evidence in different contexts revealed that receiving social support was generally beneficial for psychosocial health [26,27,28,29,30], although there were some inconsistent results [17], and also that providing it might be even more beneficial than receiving it [31,32].

Finally, although from the SIT perspective traditionally personal and social identities are considered as the end points of a continuum [33], identity theory research has also analyzed the relationships of social identity with personal identity strength e.g., [34]. From this perspective [35], people have different components of self-linked to each of the group role behavioral sets that they perform. The self, or personal identity, can be seen as a collection of identities that reflects the multiple roles that a person occupies in groups to which he/she belongs [35,36]. Even if personal and social identity can be theorized and measured as separate, the fact of being strongly intertwined structures allows for the permeability of personal and social identities [37]. Furthermore, it has been suggested [5,38] “that a strong sense of ‘me’ flows from a strong sense of ‘us’” ([5], p. 32). Consistent with this idea, and in accordance with SIT, research has shown [5,38] that a sense of shared social identity—as a result of group membership—has the ability to make group members feel capable and with personal control over their lives, through the development of a sense of agency, self-efficacy and power [5], with positive consequences for their health and well-being. In sum, these results show that social group memberships generate personal benefits through greater perceived personal control [38].

**Figure 1:**
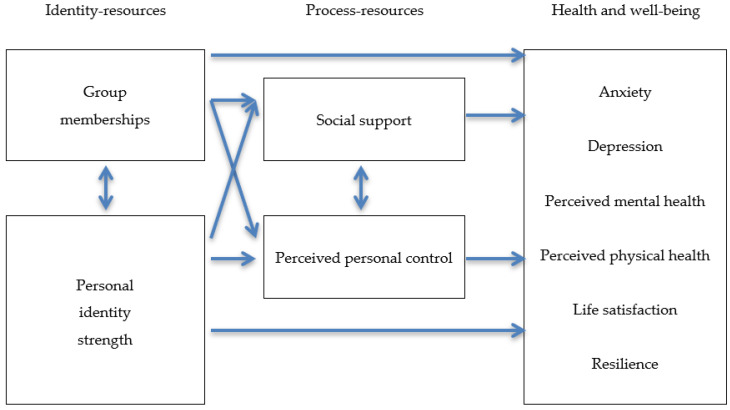
Proposed model on the relationships between identity-resources, process-resources and health and well-being outcomes. Source: Adapted from [5] (p. 347).

The Social Cure approach proposed by [5,39] posits a three component model: social identity and identification factors (e.g., multiples identities, multiple group membership, social identity continuity, personal identity strength, and so on), process factors (e.g., group norms, social support, perceived discrimination, perceived personal control, and so on), and health and well-being factors (such as depression, anxiety, stress, resilience, affect, personal self-esteem, life satisfaction or general health) related with both antecedent factors (social identity and process) ([5], see p. 347 for an overview of the model). Based on this model, our study explores whether: (1) group memberships (specifically, membership continuity and new memberships) and personal identity strength, considered as identity-resources derived from group social identities, and (2) social support (received and provided) and perceived personal control, considered as process-resources derived from the identity-resources, are related to well-being and psychological health in the confinement experience during the COVID-19 pandemic. Figure 1 summarizes the proposed model.

In the absence of previous studies from this theoretical perspective on the effects of confinement, and based on the propositions of the SIT and the Social Cure model on the specified relationships between these constructs, as well as on the available empirical evidence on their combined effects on health, we formulated the following research questions:

RQ1: How will group memberships be related to psychological health and well-being during the confinement experience?

RQ2: How will the strength of personal identity be related to psychological health and well-being during the confinement experience?

RQ3: How will social support received and social support provided be related to psychological health and well-being during the confinement experience?

RQ4: How will perceived personal control be related to psychological health and well-being during the confinement experience?

## 2. Materials and Methods

### 2.1. Ethical Considerations

This study has been approved by the Research Ethics Committee of Universidad Rey Juan Carlos (Madrid, Spain), record number 0604202009320, dated 08-04-2020, and meets all ethical and legal standards applicable to research of this survey modality.

### 2.2. Participants

The participants were 421 inhabitants from the Region of Madrid, 289 women (68.6%) and 128 men (30.4%), mean age 45.38 years (SQ = 15.40, range = 17–89). The most frequent level of studies completed was a bachelor’s degree (32.1%), followed by a master’s degree (18.1%) and a high school diploma (17.6%).

### 2.3. Instruments

Participants completed a questionnaire via the Qualtrics platform, that included socio-demographical data and self-report measures related to the study variables. The socio-demographic and occupational variables were evaluated by means of ad hoc items, related to age, gender, current occupational status, educational level, maintaining or not a couple relationship during confinement, having or not having children, belonging to a COVID-19 risk group, type of work during confinement, and being or not a health professional.

#### 2.3.1. Group Membership 

This variable was measured with the Exeter Identity Transition Scales (EXITS) [40]. The scale has two dimensions: Membership (or Social Identity) Continuity and New Membership. Membership Continuity: four items, scored on a 7-point Likert response format ranging from 1 (completely disagree) to 7 (completely agree). The time frame is before confinement. An example item is: “Before the confinement start: …I belonged to many different groups”. New Memberships: four items, with the same 7-point Likert response format. “During the period of confinement: …I’ve joined one or more new groups” is an example item. The internal consistency was adequate for both dimensions (α = 0.94 and α = 0.81, respectively). Higher scores indicated higher levels of group membership.

#### 2.3.2. Personal Identity Strength 

This scale [41] consisted of five items scored on a 7-point Likert response format (1 = completely disagree, 7 = completely agree). Item example, “I know what I want in life”, and the reliability was good. The internal consistency was α = 0.76.

#### 2.3.3. Social Support

This is a short version of a 10-item measure originally developed by [24]. The measure incorporates items designed two subscales, Received Social Support (e.g., “I know what I want in life”) and Provided Social Support (e.g., “Get the emotional support you need from others”), with 4-items each one and with 7-point Likert format, from 1 (completely disagree) to 7 (completely agree). Both subscales showed high reliability (α = 0.89 and α = 0.88, respectively).

#### 2.3.4. Perceived Personal Control 

This is measured with the scale developed by [38]; 3 items scored on a 7-point Likert response format (1 = completely disagree, 7 = completely agree). The alpha coefficient was α = 0.81. An example item is: “I feel in control of my life”.

#### 2.3.5. Anxiety 

The instrument used was the Generalized Anxiety Disorder-7 (GAD-7) [42] in its Spanish adaptation [43]. This widely used test consists of seven items, in which the subject must respond according to his agreement with one of the four possible alternatives, thinking about what happened during the last week, 0 = never; 1 = several days; 2 = more than half the days; 3 = almost every day. An example item statement is: “Feeling nervous, anxious or on edge”. Higher scores indicated higher levels of anxiety symptoms. The reliability was α = 0.91.

#### 2.3.6. Depression 

We used the Patient Health Questionnaire—Depression Scale (PHQ-9) [44] adapted to Spanish [45]. Nine items scored on a 4-point Likert response format (0 = never; 1 = several days; 2 = more than half the days; 3 = almost every day) to answer about the personal situation in the last week. An example item statement is: “Little interest or pleasure in doing things”. Higher scores indicated higher levels of depressive symptoms. Cronbach’s alpha was α = 0.88.

#### 2.3.7. Resilience 

Brief Resilience Scale (BRS) [46]: six items with a Likert scale scored on a 7-point Likert response format (1 = completely disagree to 7 = completely agree) to answers for items such as “I tend to bounce back quickly after hard times”. The internal consistency was α = 0.80.

#### 2.3.8. Life Satisfaction 

This parameter was measured by the Satisfaction with the life Scale (SWLS) [47], in its Spanish version [48]. This scale consisted of five items that were scored on a 7-point Likert-type scale (1 = completely disagree, 7 = completely agree). An item example is: “The kind of life I lead is similar to the kind of life I always dreamed of leading”. Cronbach’s alpha was α = 0.85.

#### 2.3.9. Mental Health and Perceived Physical Health

Two measures developed by [49] were used. The Mental Health one has four items, with a dichotomous response (Yes/No) and people should think about what happened last week to answer questions like: “Have you enjoyed life most of the time?” Cronbach’s alpha was α = 0.76. The questions of the Perceived Physical Health Scale have a different answer format for each of the three items, and score was calculated as an index. An example item is “Do you often have pain problems?”

### 2.4. Procedure

In Spain, population lockdown took effect at 00:00 on Sunday, 15 March 2020. Due to the isolation circumstances in which the research team conducted this study, a virtual snowball sampling was used. For the sampling to produce significant monitoring data, access to subjects was ensured from six personal and two professional networks.

Participants completed a self-report questionnaire on a Qualtrics online platform which they accessed via a web link, after being informed of the study and giving their consent to be included in it. The data collection was carried out from 13 to 20 April 2020, after one month of confinement.

### 2.5. Statistical Analysis

For statistical procedures, Statistical Package for Social Sciences (SPSS) v. 26 (IBM Corp., Armonk, NY, USA) was used. The Kolmogorov-Smirnov normality test was used to verify data distribution. Results for normality test failed, and therefore nonparametric contrast tests were conducted.

To verify the relation between identity, social support and well-being measures, Spearman’s rank correlation coefficient test was used. To assess differences on these non-normally distributed continuous variables among socio-demographic characteristic groups, analyses of covariate were conducted using Mann-Whitney U test for 2 independently sampled groups and Kruskal-Wallis H test for 3 or more groups using post-hoc Dunn test with Bonferroni adjustment. Mann-Whitney U tests and Kruskal-Wallis tests results are expressed as median (range).

## 3. Results

### 3.1. Descriptive Results

The socio-demographic characteristics of our study sample are described in Table 1.

Firstly, we offer the most significant differences in relation to gender (Table 2), age (Table 3), educational level (Table 4), working and occupational status (Table 5), people who were (or not) in a relationship in confinement (Table 6), and membership of a COVID-19 risk group.

Regarding gender differences (Table 2), the Mann-Whitney test indicated that the provided social support was greater for females than for males, although females also showed higher values for anxiety and depression. Males scored higher in resilience and physical health.

With regard to age group differences, the Kruskal-Wallis H test performed showed a significant difference in New Memberships (*χ*^2^ (6, *N* = 421) = 14.35, *p* = 0.026). The most significant post hoc results showed (Table 3) worse results for the younger age groups (less than 39 years). Notably, group 2 (20–29 years old) presented worse general indicators, with higher levels of anxiety and depression and lower levels of mental health than participants over 40, and lower levels of perceived physical health, resilience and life satisfaction than those in group 6 (60–69 years). Similarly, group 3 (30–39 years) presented greater anxiety and worse mental health than the participants over 60 years old, the latter result also obtained by those of 18–19 years old.

In reference to the educational level, the Kruskal-Wallis H test reported significant differences between groups in Continuity in Membership (*χ*^2^ (7, *N* = 421) = 18.56, *p* = 0.010) and New Memberships (*χ*^2^ (7, *N* = 421) = 21–68, *p* = 0.003). As can be seen in Table 4, participants with higher levels of education showed better general indicators. Specifically, those with doctoral degrees expressed lower levels of anxiety and depression than high school graduates, and higher levels of mental health and resilience than those with primary education. While those with a master’s degree reported greater group membership continuity than high school graduates, and more new group memberships than those with a bachelor’s degree.

As shown in Table 5, people who were working during the period of confinement (teleworking or face-to-face) presented greater membership continuity, life satisfaction, resilience and mental health than people who didn’t work (temporary suspension of contract, sick leave, ERTE or inactivity due to confinement). Moreover, people who didn’t work expressed higher levels of depression that people who were in working in confinement.

Regarding occupational status, the most significant results showed that retirees experienced lower levels of anxiety and depression than part-time workers and students, better mental health than part-time workers, the unemployed, COVID-19 unemployed, and students, and also less depression than the latter three groups. In addition, students showed less resilience than retirees, worse mental health and greater depression than self-employed and full-time workers, and greater anxiety than the latter. The results shown by the students are consistent with those already mentioned for the younger age groups (Table 3). Compared to other professionals (O), health professionals (HP) reported that they developed more new group memberships, showed greater personal identity strength, provided more social support, and had more perceived personal control.

The results in Table 6 show that people who were in a relationship in confinement showed lower levels of depression and better mental health. In turn, people with children showed less anxiety and depression, and also displayed higher personal identity strength, life satisfaction, resilience, mental health and physical health. Finally, people who belonged to a COVID-19 risk group only reported worse perceived physical health (*Mdn* = 7.29, *IQR* = 8.29–5.78) than those who did not belonged (*Mdn* = 7.86, *IQR* = 8.86–6.29), *U* = 14481.50, *p* = 0.021).

### 3.2. Correlational Results

As shown in Table 7, maintenance of memberships before quarantine was correlated with the building of new ones during quarantine (*ρ* = 0.34, *p* < 0.01). Membership continuity was associated with identity strength (*ρ* = 0.20, *p* < 0.01), but new memberships were not correlated with it. Meanwhile, received social support correlated with membership continuity (*ρ* = 0.22, *p* < 0.01) and with personal identity strength (*ρ* = 0.30, *p* < 0.01). And provided social support was positively associated with membership continuity (*ρ* = 0.29, *p* < 0.01), new memberships (*ρ* = 0.12, *p* < 0.05) and highly with personal identity strength (*ρ* = 0.41, *p* < 0.01). Moreover, perceptions of received and provided social support were strongly correlated (*ρ* = 0.56, *p* < 0.01). Spearman’s rank correlation coefficients related to perceived control are also presented in Table 2. Personal identity strength showed a significant association (*ρ* = 0.28, *p* < 0.01), and also received social support (*ρ* = 0.24, *p* < 0.01) and provided social support (*ρ* = 0.31, *p* < 0.01). Correlations regarding relationships group and identity-resources measures and health and well-being perceptions are also shown in Table 2. Membership continuity was correlated with resilience (*ρ* = 0.15, *p* < 0.01). Personal identity strength was positively associated with resilience (*ρ* = 0.30, *p* < 0.01), life satisfaction (*ρ* = 0.30, *p* < 0.01), mental health (*ρ* = 0.21, *p* < 0.01) and perceived physical health (*ρ* = 0.17, *p* < 0.01) and negatively with anxiety (*ρ* = −0.11, *p* < 0.05) and depression (*ρ* = −0.18, *p* < 0.01). New identities showed no associations with well-being. Meanwhile, received social support was positively associated with resilience (*ρ* = 0.18, *p* < 0.01), life satisfaction (*ρ* = 0.31, *p* < 0.01), mental health (*ρ* = 0.22, *p* < 0.01) and perceived physical health (*ρ* = 0.21, *p* < 0.01) and negatively with anxiety (*ρ* = −0.12, *p* < 0.05) and depression (*ρ* = −0.17, *p* < 0.01). Whereas provided social support was positively associated with resilience (*ρ* = 0.21, *p* < 0.01), life satisfaction (*ρ* = 0.27, *p* < 0.01), mental health (*ρ* = 0.16, *p* < 0.01) and perceived physical health (*ρ* = 0.16, *p* < 0.01) and negatively with anxiety (*ρ* = −0.10, *p* < 0.05). Lastly, perceived control was positively associated with resilience (*ρ* = 0.29, *p* < 0.01), strongly with life satisfaction (*ρ* = 0.57, *p* < 0.01), mental health (*ρ* = 0.35, *p* < 0.01) and perceived physical health (*ρ* = 0.43, *p* < 0.01) and negatively with anxiety (*ρ* = −0.34, *p* < 0.01) and depression (*ρ* = −0.36, *p* < 0.01).

### 3.3. Clustering and Comparative Results

We conducted additional analysis in order to delve deeper into the relationships between received and provided social support during the pandemic, perceived personal control and personal identity strength and the different health and well-being factors. To this end, these variables were recoded in three clusters or groups as follows: level 1 or low (equal to or below the 25th percentile), level 2 or medium (above the 25th and below the 75th percentile), and level 3 or high (equal to or above the 75th percentile). To study the differences between the three clusters of each variable, the Kruskal-Wallis H test and the Dunn-Bonferroni post hoc test were performed.

With respect to differences between levels of received social support, the Kruskal-Wallis H test showed significant differences in life satisfaction (χ^2^ (2, N = 421) = 35.03, *p* < 0.001), depression (χ^2^ (2, N = 421) = 9.01, *p* = 0.011), mental health (χ^2^ (2, N = 421) = 15.55, *p* < 0.001), resilience (χ^2^ (2, N = 421) = 10.02, *p* = 0.007) and physical health (χ^2^ (2, N = 421) = 17.65, *p* < 0.001). The results of the Dunn-Bonferroni post hoc test pointed out that people who experienced a high level of received social support had greater mental health (*p* < 0.05), and physical health (*p* < 0.01) than those who had a low or medium level. They also expressed greater resilience (*p* < 0.01) and lower depression (*p* < 0.05) than the low-level group. In addition, the medium group had greater life satisfaction (*p* < 0.01) than those with low level. As for the differences in the levels of provided social support, our data reported significant differences in life satisfaction (χ^2^ (2, N = 421) = 25.15, *p* < 0.001), mental health (χ^2^ (2, N = 421) = 8.08, *p* = 0.018), resilience (χ^2^ (2, N = 421) = 15.57, *p* < 0.001) and physical health (χ^2^ (2, N = 421) = 8.69, *p* = 0.013). The Dunn-Bonferroni post hoc test showed that those with a high level of provided social support had greater life satisfaction (*p* < 0.001), mental health (*p* < 0.05), resilience (*p* < 0.001) and physical health (*p* < 0.05) than the group of low level. Medium level group presented greater life satisfaction (*p* < 0.01) and resilience (*p* < 0.05) than low level group.

With regard to the differences between the three groups of perceived personal control, the performed test reported significant differences in life satisfaction (χ^2^ (2, N = 421) = 115.56, *p* < 0.001), anxiety (χ^2^ (2, N = 415) = 43.87, *p* < 0.001), depression (χ^2^ (2, N = 410) = 52.04, *p* < 0.001), mental health (χ^2^ (2, N = 417) = 45.60, *p* < 0.001), resilience (χ^2^ (2, N = 418) = 31.97, *p* < 0.001), and physical health (χ^2^ (2, N = 421) = 72.42, *p* < 0.001). The Dunn-Bonferroni post hoc test showed that those with a high level of perceived personal control had greater life satisfaction (*p* < 0.001), mental health (*p* < 0.001), resilience (*p* < 0.001) and physical health (*p* < 0.001), and lower levels of anxiety (*p* < 0.001) and depression (*p* < 0.001) than the group of low level. Moreover, presented greater life satisfaction (*p* < 0.001), mental (*p* < 0.05) and physical (*p* < 0.001) health and lower depression (*p* < 0.05) than the group of medium level. In addition, the group of medium level showed greater life satisfaction (*p* < 0.001), mental health (*p* < 0.001), resilience (*p* < 0.001) and physical health (*p* < 0.001) and lower anxiety (*p* < 0.001) and depression (*p* < 0.001) levels than the group of low level.

Finally, attending to the differences between the levels of personal identity strength, the results indicated the existence of significant differences in life satisfaction (χ^2^ (2, N = 421) = 38.39, *p* < 0.001), depression (χ^2^ (2, N = 410) = 12.69, *p* = 0.002), mental health (χ^2^ (2, N = 417) = 18.44, *p* < 0.001), resilience (χ^2^ (2, N = 418) = 31.43, *p* < 0.001) and physical health (χ^2^ (2, N = 421) = 10.64, *p* = 0.005). The post hoc tests reported than those with high level of personal identity strength had greater life satisfaction (*p* < 0.001), resilience (*p* < 0.001), mental (*p* < 0.001) and physical health (*p* < 0.01) and lower depression (*p* = 0.001) than those belonging to the low group. Also presented greater resilience (*p* = 0.001) than the medium level group. Moreover, the medium level group had greater life satisfaction (*p* < 0.001) and mental health (*p* < 05) than the low-level group.

## 4. Discussion

In relation to the first objective of our study, our preliminary results showed that women had poorer mental health (anxiety and depression) than men, who showed better physical health and greater resilience. Prior evidence on gender differences in relation to the psychological consequences of quarantine was inconclusive, with some studies [50] showing a greater negative psychological impact on women, while others [11,51] found no difference. Our results did show a higher incidence in women, although it would be necessary to know the history of psychiatric illness in order to conclude that these worse mental health indicators were related to the experience of confinement. Meanwhile, our data showed that women were providers of social support to a greater extent than men. This result was consistent with the evidence for women’s greater ability to provide social support—as gender (femininity) not as sex [52]; however, by showing higher levels of depression than men, it appears that the social support exchanged between women, and that received from men, was not sufficient to buffer the negative effect of confinement stressors. This interpretation is consistent with the evidence suggesting [53] that women are better providers of social support to men than men are to women. However, this result does not confirm the prediction [54] that the modalities of social support (empathy, active coping assistance, and role modeling) provided by experientially similar others—i.e., women—be efficacious in alleviating the psychological impacts of stressors.

Regarding age groups, the worst mental health (anxiety and depression) and the lowest well-being (life satisfaction) of the 20–29 year-old group and the students can be highlighted. This result is also consistent with prior evidence; for instance [50] found that the 16–24 year age group suffered the most negative psychological impact in a quarantine situation, and [51], in a study conducted in China in the context of COVID-19, have found that the younger population perceived more impacts of the epidemic outbreak (changes over living situations or emotional control), negative coping style and had higher level of psychological distress. The poorer mental health and lower life satisfaction of young people, as well as of students, can be explained by the disruption of their lifestyle by confinement, at an age where relationships and social contact are high valued. In addition, the economic effects of COVID-19 on employment may further affect young people’s precarious employment and career opportunities, increasing their frustration.

Regarding educational level, people with doctoral degree show greater continuity of membership and more new memberships than people with close educational levels (high school and bachelor’s degrees), as well as better mental health, lower anxiety and depression, and greater resilience than those with primary education and elementary school degree. Consistent with these results, there is broad evidence (e.g., [5,24,55]) that people with high (perceived) socio-economic status (assessed in terms of level of education) or with high (perceived) group status have more social capital and it is beneficial for their well-being and health.

People who had a relationship and cared for children during confinement manifest better mental health and higher strength of identity, life satisfaction, resilience and physical health. Data from China’s population in the early stages of the COVID-19 quarantine [51] showed that unmarried people were more aware of the impacts of the epidemic outbreak and had a higher level of psychological distress. These data seem to indicate that affective and family relationships can play a buffering role of confinement stressors. The results also indicated that continuing to work (face-to-face or teleworking) was associated with better indicators of social identity and mental health compared with those who did not work for any reason. In this case, it appears that the potential stress from working under the conditions imposed by the pandemic was not experienced or added to the confinement stressors. Rather, it can be interpreted that group membership continuity (i.e., identity continuity) facilitated by work activity is related to a perception of less life disruption, and consequently, to better mental health and greater resilience and vital satisfaction [6]. It is also possible that downward social comparisons of people in working with those who lost their jobs or were infected by COVID-19, may have facilitated their perception of psychological well-being [56].

Regarding retirees, the results also seem to underline the importance of identity, as is the case for working people. In this regard, although they no longer worked, their mental health indicators were better (along with those of full-time employees) than those of most occupational groups. This interpretation is consistent with previous data [5,57] indicating that new group memberships and identification as a retiree play a protective role and has positive effects on well-being and mental health.

As for our second objective and the four research questions that we asked, our results allow us to give a preliminary answer to all of them. Identity-resources showed a significant relationship between them, so that membership continuity was associated with new memberships during confinement. This result may be relevant, as it indicates that in such a life disruptive experience the identities associated with group memberships were maintained and could facilitate new identities through memberships that were necessarily largely adopted without face-to-face contact. This result is consistent with SIT’s postulates [5,21] and Self-Categorization Theory (SCT) [22] that the (cognitive) perception of group membership is sufficient to create a group identity. As SCT posit [22,58], group membership and social category-based self-conceptualization are motivated by uncertainty reduction [59]. Thus, contextual uncertainty created by confinement and the COVID-19 pandemic could be reduced entering new groups, adopting new identities and by group action, although within the limits imposed by social distancing and quarantine measures.

A significant relationship was also found between membership continuity and personal identity strength, which coincides with SIT’s approaches that defend the links between social identity and personal identity [35,36] and permeability of both [37]. The correlation between the new memberships and personal identity strength was not significant, but this may be due to the relatively short time that has passed since the adoption of new group identities (less than a month), so it will be relevant to analyze whether this relationship is reinforced over time. In sum, our results show that participants had both identity-resources during the confinement, and it is important to check, as our model based on the Social Cure approach [5] proposes, if they are related to process-resources and, especially, to well-being and health perceptions.

With regards to social support, a significant relationship was found between support received and support provided. This result is relevant, because prior evidence indicates [60] that both occur when the size of social network is large, which again is associated with group membership, since a positive relationship was found between continuity and social support received and given, and new membership and social support provided during confinement. Our results are coincident with prior research founding that shared social identities and group memberships are social support exchange opportunities for receiving, giving, and benefit from both social actions [5,24,25]. In addition, personal identity strength was positively related to received and provided social support and perceived personal control. In turn, both types of social support were significantly related to perceived personal control, which is consistent with the previous literature [61,62,63]. Thus, it can be concluded that social support exchange with people who define themselves in terms of shared social identity are related with the development of a sense of control, which constitute important psychological resources for people [5,23].

Our model based on the Social Cure approach [5] suggested that both types of social and personal resources (identity-resources and process-resources) would be related to perceived well-being and health. Correlational analyses and, especially, additional analyses conducted clustering in low, medium and high levels of identity and process resources, confirm the relationships between strength of personal identity, social support received and provided, and perceived personal control and health and psychological well-being consequences experienced during the COVID-19 pandemic. The clearest indicator of these relationships is resilience, since all resource variables, except for new memberships, are positively and significantly related to it. These results are reinforced by the significant differences found in the greater resilience of groups with high levels of social support received and provided, perceived personal control, and strength of personal identity in relation to groups with medium and low levels of these three resources. These results are consistent with previous evidence that identifies multiple group memberships [64], strength of personal identity [65], social support received [66], especially in people exposed to trauma [67], and perceptions of personal control [68] as antecedents to resilience. Overall, our results are aligned with the perspective that postulates social identity as a basis for resilience, both individual [5] and collective resilience [69], and have important implications for the design of psychosocial interventions that foster group membership and participation, specially to coping with potential future confinement experiences in cases of new outbreaks of the COVID-19. For their part, anxiety experienced during confinement was less, perceived mental health was greater, and life satisfaction was higher for people who felt personal identity strength, received and provided social support, and perceived personal control. These results are also consistent with extensive prior evidence [5,17,18,19], and are relevant for future interventions, as discussed below. Similar results were found for depression, although in this case the social support provided was not significantly related. Finally, people who pertained to the cluster of high levels of provided and received social support, personal identity strength, and perceived personal control experienced in general greater mental health and life satisfaction and better overall physical health than groups of medium and low level, which is also consistent with prior evidence [5,38,41].

### 4.1. Practical Implications

The main implications of our study concern the design of psychosocial interventions. For instance, the team of Haslam et al. [5] has successfully implemented a social intervention program labeled Groups 4 Health that develops the Social Identity Model of Identity Change (SIMIC) [39], applied to life transitions as retirement. In a similar vein, our model proposal and the results obtained can be useful for the design of strategies and psychosocial interventions in the sense of strengthening social networks and the potential social support derived from them. Given the strong link between social relations, support and mental health [17,63], actions aimed at strengthening interpersonal relations and social networks through, for example, support groups, community initiatives or networks of people who share similar characteristics (of studies, profession, interests, etc.) [70], can be very effective in providing social coping and resilience resources that increase the personal resources of those who experience, for example, post-traumatic stress following confinement.

Interventions designed with the Groups 4 Health model to address problems of social isolation or loneliness, stress, anxiety and depression have proven effective [71], so that its adaptation to address the potential psychological consequences of confinement appears promising. This proposal for group-based interventions is even more relevant if we take into account the warnings of health authorities regarding potential outbreaks of COVID-19 in the coming months.

### 4.2. Limitations and Future Research

This study was conducted with a non-probabilistic sample and using a measure of the study variables one month after the start of the confinement. Consequently, only relationships between variables can be established, so our aim is for future studies to include a greater number of temporary measures with subsequent follow-ups in order to identify causal relationships between variables. In this sense, longitudinal studies may allow to test the potential mediating role of process-resources in the relationship between identity-processes and factors related to health and well-being [5]. Likewise, the participants were residents of the Region of Madrid and the sample size is relatively small, so the results cannot be generalized to the Spanish population. However, given that Madrid was one of the European regions where the COVID-19 was most virulent, our results are relevant for understanding the experiences of citizens and their perceived levels of well-being and health in a situation of extreme alarm. Future research, as well as potential interventions, should test whether resources based on social and personal identity and group membership, as well as the process resources provided by them (social support and perceived control), can play a protective (buffering) role for well-being and health in disruptive situations such as that triggered by the COVID-19 pandemic. If future threats of virus outbreaks or similar health crises occur, this knowledge can be of great value and use in helping people to cope and overcome them with as little harm to their well-being and health as possible.

## 5. Conclusions

In short: our results provide a first overall answer to our research questions: identity-resources and process-resources associated with them have positive relationships with the levels of well-being and health experienced during confinement. Furthermore, as far as we know, this is the first time that the Social Cure model [5,38] is used in a sample of the Spanish population, and our results may complement those obtained by other studies carried out in Spain during the COVID-19 pandemic (i.e., [72,73,74]). Finally, our study may also contribute to the design of interventions based on this model, as we discussed in the prior section.

## Figures and Tables

**Table 1:** Frequencies and percentages of the socio-demographic variables considered.

Variables and Levels	Freq.	%	Variables and Levels	Freq.	%
Gender			Couple’s relationship		
Males Females	128 289	30.4 68.6	Yes No	296 125	70.3 29.7
Risk group Yes No	109 312	25.9 74.1	Having children Yes No	228 193	54.2 45.8
Age group 18–19 (1) 20–29 (2) 30–39 (3) 40–49 (4) 50–59 (5) 60–69 (6) Over 70 (7)	15 73 60 99 88 69 17	3.6 17.3 14.3 23.5 20.9 16.4 4	Educational background Primary Baccalaureate Medium grade/PT Technical engineering Bachelor’s degree Master PhD Others	12 74 50 37 135 76 33 4	2.9 17.6 11.9 8.8 32.1 18.1 7.8 1.0
Work in confinement Work-presence Teleworking Suspended by COVID-19 ERTE * COVID-19 sick leave Sick leave (other) Not working at home (but employed) Others	42 166 6 18 8 7 14 36	10 39.4 1.4 4.3 1.9 1.7 3.3 8.6	Occupational status Full-time employee Part-time employee Self-employee Unemployed Unemployed by COVID-19 Retired Student Other	212 13 37 27 11 52 58 11	50.4 3.1 8.8 6.4 2.6 12.4 13.8 2.6
Health professional Yes No	74 347	17.6 82.4			

* ERTE = Temporary layoff file by COVID-19.

**Table 2:** Descriptives (Md (IQR)) of the variables with significant differences by gender.

Variables	Males	Females
Provided social support	22 (24–19)	23 (25–20)
Anxiety	5 (8–2)	6 (11–3)
Depression	4 (8–2)	6 (10–3)
Resilience	29 (33–25)	27 (31–23)
Perceived physical health	7.8 (9.4–6.7)	7.2 (8.4–6.1)

**Table 3:** Descriptives (Md (IQR)) and post-hoc differences by age group.

Variables	1 (18–19)	2 (20–29)	3 (30–39)	4 (40–49)	5 (50–59)	6 (60–69)	7 (>70)	Post-hoc Differences ^1^
New memberships	5 (8–4)	7 (11–4)	6.5 (11–4)	7 (11–4)	7.5 (13–4)	5 (10–4)	4 (5.5–4)	7 < 5
Life satisfaction	23 (29–21)	20 (25–15)	22 (25.7–16.2)	25 (29–20)	22.5 (27–18)	25 (28–21)	26 (29–22)	2 < 4, 2 < 6
Anxiety	8 (10–4)	10 (16.5–6)	7 (12–5)	6 (8–2)	6 (8–3)	4 (6.7–1.2)	4 (5–1)	1 > 6, 2 > 4 to 7, 3 > 6 & 7
Depression	8.5 (12.5–4.5)	11 (17–6)	7 (10–4)	5 (8–2)	5 (9–3)	2 (6–1)	3.5 (4.7–1.2)	6 < 1 & 5, 2 > 4, 5 & 7
Mental health	7 (8–5)	5 (7–4)	7 (8–5)	8 (8–7)	7 (8–6.7)	8 (8–7)	8 (8–8)	2 < 4 to 7, 1 < 6 & 7, 3 < 6 & 7
Resilience	24 (30–22)	25 (30–20)	27 (30.7–24)	28 (33–24)	27 (31–23)	34 (31–25)	29 (33–27)	6 > 2
Perceived physical health	8.3 (9.4–8.1)	6.7 (8–6)	7.7 (8.2–6.1)	7.8 (8.8–6.2)	7.7 (8.8–6)	8 (8.8–6.8)	7.2 (7.8–6)	2 < 6

^1^ Dunn-Bonferroni post hoc test. Only significant differences between groups appear (*p* < 0.05).

**Table 4:** Descriptives (Md (IQR)) and post-hoc differences by educational level.

Variables	1 Primary	2 Baccalaureate	3 Medium Grade	4 Technical Engineering	5 Bachelor’s Degree	6 Master	7 PhD	Post-hoc Differences ^1^
Membership continuity	16 (22.2–6.5)	15.5 (20–9.7)	16 (22–11)	16 (23–12)	18 (23–13)	21 (26–15)	17 (26–12)	6 > 2
New memberships	7 (10–4)	6.5 (12–4)	6.5 (10–4)	6 (12–4)	5 (10–4)	9 (15–4.2)	9 (13.5–4.5)	6 > 5
Anxiety	7 (15–2.7)	7 (13–4)	7 (9.2–3)	7 (11–3.5)	6 (10–2)	6 (7–3)	4 (6–2.5)	7 < 2
Depression	8.5 (15.7–3.7)	8 (14–4)	6 (10–2)	6 (10–3)	5 (8–2)	6 (11–3)	3 (6–1.5)	7 < 2, 7 < 1
Mental health	5.5 (7–4)	7 (8–5)	7 (8–5)	7.5 (8–5.2)	8 (8–6)	7 (8–6)	8 (8–7)	7 > 1
Resilience	22 (25.7–16)	26 (30–22)	28 (32–24)	28 (33–24)	27 (33–24)	29 (33–23)	31 (34–24.5)	7 > 1

^1^ Dunn-Bonferroni post hoc test. Only significant differences between groups appear (*p* < 0.05).

**Table 5:** Descriptives (Md (IQR)) and post-hoc differences by working and occupational status.

Working or Not During Confinement								
Variables	Working				Not working			
Membership continuity	19 (25–13)				15 (21–11)			
Life satisfaction	24 (28–19)				22 (26–17)			
Depression	5 (8–2.25)				6 (13–3)			
Mental health	7 (8–6)				7 (8–5)			
Resilience	28 (33–24)				26 (30–22)			
**Occupational Status**								
	**1 Full-Time**	**2 Part-Time**	**3 Self-Employed**	**4 Unemployed**	**5 Unemployed by COVID**	**6 Retired**	**7 Student**	**Post-Hoc Differences ^1^**
Life satisfaction	24 (28–19)	19 (24–16)	23 (26.5–17)	20 (26–16)	20 (23–12)	25 (28.7–21.2)	21.5 (26–18)	ns
Anxiety	6 (8–2)	7 (16–5.5)	5.5 (11–3)	7.5 (15.2–3.7)	10 (13–3)	4 (5.2–2)	10 (15.2–5)	6 < 2 & 7, 1 < 7
Depression	5 (8–3)	9 (13.5–5)	5 (9–2.2)	9 (15.2–4)	10 (18–3.7)	3 (6–1)	11 (16–6)	6 < 2, 4, 5 & 7, 7 > 1 & 3
Mental health	7 (8–6.25)	7 (7–5)	7 (8–6)	7 (8–4)	6 (7–5)	8 (8–7)	5 (7–4)	7 < 1, 3 & 6, 6 > 2, 4 & 5
Resilience	28 (32–24)	25 (30–22)	29 (32.7–25)	26 (32–23)	22 (29–22)	30 (33–25)	25 (30–20)	6 > 7
**Health Professionals and Others**								
	**Health Professionals**				**Other Professionals**			
New membership	9 (9–5)				6 (6–4)			
Personal identity strength	30.5 (30.5–28)				29 (29–26)			
Provided social support	24 (24–20)				22 (22–19)			
Perceived Control	16 (16–13)				14 (14–11)			

^1^ Dunn-Bonferroni post hoc test. Only significant differences between groups appear (*p* < 0.05).

**Table 6:** Descriptives (Md (IQR)) of the variables with significant differences for “having or not a relationship during confinement” and “having or not children”.

Variables	In Couple	Not in Couple
Depression	5 (10–2)	6 (11–3)
Mental health	7 (8–6)	7 (8–5)
	**With children**	**Without children**
Personal identity strength	30 (33–28)	29 (32–25)
Life satisfaction	25 (29–20)	22 (25–17)
Resilience	29 (33–24)	26 (30–22)
Mental Health	8 (8–7)	7 (8–5)
Physical health	7.86 (8.86–6.29)	7.29 (8.29–6.14)

**Table 7:** Descriptive Statistics and Spearman’s rank correlation coefficient for Quantitative Study Variables.

	*n*	Mdn	Min-Max IQR	1	2	3	4	5	6	7	8	9	10	11
1. Membership Continuity	421	18.00	4.00–28.00 10.75	(0.94)										
2. New Memberships	421	6.00	4.00–28.00 7.00	0.34 **	(0.81)									
3. Personal Identity Strength	421	30.00	6.00–35.00 6.00	0.20 **	0.07	(0.76)								
4. Received Social Support	421	22.00	4.00–28.00 8.00	0.22 **	0.05	0.30 **	(0.89)							
5. Provided Social Support	421	22.00	4.00–28.00 4.00	0.29 **	0.12 *	0.41 **	0.56 **	(0.88)						
6. Perceived Personal Control	421	15.00	3.00–21.00 6.00	0.06	0.04	0.28 **	0.24 **	0.31 **	(0.81)					
7. Anxiety	415	6.00	0.00–21.00 6.00	0.04	0.05	−0.11 *	−0.12 *	−0.10 *	−0.34 **	(0.91)				
8. Depression	410	6.00	0.00–26.00 7.00	−0.01	−0.01	−0.18 **	−0.17 **	−0.09	−0.36 **	0.77 **	(0.88)			
9. Resilience	418	27.00	6.00–42.00 9.00	0.15 **	0.08	0.30 **	0.18 **	0.21 **	0.29 **	−0.49 **	−0.46 **	(0.80)		
10. Life Satisfaction	421	23.00	5.00–35.00 9.00	0.09	0.02	0.30 **	0.31 **	0.27 **	0.57 **	−0.35 **	−0.42 **	0.34 **	(0.85)	
11. Mental Health	417	7.00	4.00–8.00 2.00	0.08	0.06	0.21 **	0.22 **	0.16 **	0.35 **	−0.68 **	−0.78 **	0.45 **	0.42 **	(0.76)
12. Perceived Physical Health	409	7.71	1.71–11.00 2.57	0.10	0.08	0.17 **	0.21 **	0.16 **	0.43 **	−0.49 **	−0.58 **	0.37 **	0.37 **	0.52 **

* *p* < 0.05, ** *p* < 0.01, Cronbach’s Alphas are shown in the diagonal, except for Perceived Physical Health, that has been calculated as an index.
